# A GRU-KAN Surrogate Model with Genetic Algorithm Uniform Sampling for Active Magnetic Bearings–Rotor Critical Speed Prediction

**DOI:** 10.3390/s25185680

**Published:** 2025-09-11

**Authors:** Jiahang Cui, Jianghong Li, Feichao Cai, Zhenmin Zhao, Yuxi Liu

**Affiliations:** 1 School of Power and Energy, Northwestern Polytechnical University, Xi’an 710129, China; cui275228896@mail.nwpu.edu.cn (J.C.); liuyuxi0318@163.com (Y.L.); 2 Science and Technology on Altitude Simulation Laboratory, Mianyang 62100, China; 3 National Key Laboratory of Science and Technology on Advanced Light-Duty Gas-Turbine, Xi’an 710129, China; 4 National Elite Institute of Engineering, Northwestern Polytechnical University, Xi’an 710129, China; 2022160894@mail.nwpu.edu.cn

**Keywords:** AMB–rotor, dynamic model, surrogate model, GRU-KAN, critical speed

## Abstract

With the development of active magnetic bearings (AMBs) toward higher speeds, understanding high-speed rotor dynamics has become a crucial focus in AMB research. Traditional finite element modeling (FEM) methods, however, are unable to rapidly and comprehensively uncover the complex interplay between controller parameters and dynamic behavior. To address this limitation, a surrogate modeling approach based on a hybrid gated recurrent unit–Kolmogorov–Arnold network (GRU-KAN) is introduced to mathematically capture the effects of coupled control gains on rotor dynamics. To enhance model generalization, a genetic algorithm-driven uniform design sampling strategy is also implemented. Comparative studies against support vector regression and Kriging surrogates indicate a higher coefficient of determination ($R^{2}=0.9887$) and lower residuals for the proposed approach. Experimental validation across multiple controller parameter combinations shows that the resulting machine learning surrogate predicts the critical speed with a mean absolute error of only 38.51 rpm and a mean absolute percentage error of $1.56\times 10^{-1}\%$, while requiring merely $1.14\times 10^{-4}$ s per evaluation—compared to 201 s for traditional FEM. These findings demonstrate the surrogate’s efficiency, accuracy, and comprehensive predictive capabilities, offering an effective method for rapid critical speed estimation in AMB–rotor systems.

## 1. Introduction

### 1.1. Background

AMBs are widely employed in systems such as torque gyroscopes, flywheel energy storage devices, blowers, centrifugal compressors, and other electric engines [1]. Early AMB research focused on manufacturing, packaging, and topology design, yielding notable performance test results [2]. As an open-loop unstable nonlinear mechatronic system, the AMB–rotor assembly requires feedback control to ensure stable operation, while the influence of the rotor dynamics must not be neglected [3]. With recent advances in computational power, models have progressively evolved toward multidisciplinary and multiparameter coupling [4,5], furnishing powerful theoretical tools for the more accurate characterization of dynamic behavior.

### 1.2. Literature Review

#### 1.2.1. Dynamic Modeling Methods for AMB–Rotors

Many researchers have investigated AMB–rotor dynamic modeling. In [6], FEM theory was used to derive MATLAB 2021a-computed and workbench-simulated dynamic models of both rigid and flexible AMB–rotors supported by AMBs, comparing modal frequencies and mode shapes under varying equivalent stiffness; the study showed that tuning AMBs’ stiffness shifts natural frequencies and thus mitigates vibration. In [7,8], an algorithm for the synchronous identification of rotor unbalance and residual AMB parameters was proposed. This algorithm was developed based on Timoshenko beam theory, the finite element method, and a misalignment approach. The efficacy of the proposed algorithm was demonstrated through experimental testing, which yielded enhanced diagnostic accuracy and robustness. In [9], the design, construction, and modeling of a flexible rotor AMB test rig were detailed: by integrating conventional rotor dynamics analysis, FEM, and experimental validation, a comprehensive system model was established, proving that precise modeling coupled with model-based controller design enables the effective control of flexible rotors. In [10], a nonlinear dynamic model coupling the AMB and backup bearing was developed, systematically incorporating contact stiffness, sliding/rolling friction, aerodynamic damping, eddy current losses, and rotor geometric irregularity. This provided a high-fidelity description of the transient dynamics during rotor delevitation. In [11], the dynamic stiffness of radial AMBs in a magnetic-suspension molecular pump was analyzed and measured via combined frequency- and time-domain experiments, yielding detailed insights into bearing dynamic characteristics. In [12], a disturbance compensation control strategy for rotational-speed standard devices using an AMB force compensation system was proposed: by analyzing rotor unbalance dynamics and linearizing and decoupling the AMB force model across radial degrees of freedom, a PI-based force feedback controller was designed and validated on a semi-physical simulation platform, demonstrating the effective suppression of static, step, and synchronous vibration disturbances. In [13], the state space-based dynamic modeling and μ-synthesis control of flexible rotors and AMBs were presented and experimentally validated. In [14], a dynamic model based on radial and tilting motions was developed; simulations indicated that increasing angular acceleration markedly reduced the vibration amplitude at the radial critical speed, enabling an AMB–motor to traverse this critical speed without additional damping. In [15], building on a rotor dynamic model with incorporated gyroscopic effects, a fractional-order PID controller was formulated and optimized via particle swarm optimization to tune both the fractional orders and gain coefficients concurrently, achieving pole placement that markedly extended the stability margins and enhanced the dynamic response under high-speed operation.

Despite advances in single-physics rotor modeling, comprehensive analysis techniques for multidisciplinary and multiparameter coupled models remain relatively underdeveloped, particularly with respect to the impact of control parameter coupling on dynamic performance. In addition, the significant computational cost of parameter coupling analysis has not been adequately addressed.

#### 1.2.2. Surrogate Model Methods

Surrogate models offer an effective route to study how control systems influence dynamic performance by rapidly constructing high-fidelity mappings between input variables and output responses [16,17,18], which endows them with unique advantages in rotor dynamics parameter identification. In [19], a hybrid surrogate model combining Kriging and Polynomial Chaos Expansion (PCE) was developed to relate crack parameters to rotor system dynamic responses, enabling an analysis of how parameter variations shape the response surface. In [20], simulated annealing enhanced particle swarm-optimized support vector regression (SVR) surrogate models and improved the high-speed spindle thermal displacement prediction accuracy while reducing the computational complexity. In [21], a KAN was applied to system dynamics prediction; the results showed superior accuracy in forecasting the rotor angular frequency and identifying system uncertainty parameters with a smaller network scale than conventional machine learning methods, demonstrating the KAN’s potential for the efficient, precise modeling of complex rotor systems. In [22], a particle swarm optimization method based on a mutation surrogate model was proposed for high-dimensional complex problems, using an initial sample selection strategy to build globally representative surrogate models. In [23], Latin hypercube sampling (LHS) was optimized to enhance surrogate models’ exploration and exploitation performance, with a staged sample selection strategy that improved the global fidelity and accuracy under limited sample sizes.

Collectively, prior studies indicate that surrogate models have advanced mechatronic system modeling and prediction through global sample selection strategies and modern machine learning techniques, thereby supporting improved model fidelity. However, conventional surrogates remain susceptible to overfitting when the training set is sparse or unevenly distributed, and their robustness and accuracy are intrinsically constrained by the coverage of the sampled design space. In particular, when the available data are limited, Latin hypercube sampling may preserve marginal uniformity yet inadequately capture the response surface curvature and higher-order interactions [24], which can lead to reduced generalization.

### 1.3. Contributions

Therefore, this paper analyzes the influence of control parameters on dynamic models and proposes a GRU-KAN model based on a genetic algorithm uniform design (GAUD), comparing and analyzing it against SVR and Kriging models to investigate in depth the coupling between control parameters and the critical speed. We also introduce a novel method for the prediction of the critical speed of the AMB–rotor. The main contributions of this paper are as follows:

1. An evolutionary uniform design guided by a composite discrepancy metric is proposed. The scheme balances space filling with factor coupling and yields representative training sets suited to control–dynamics analysis.
2. A surrogate that combines gated recurrent units with a Kolmogorov–Arnold network is formulated to capture nonlinear interactions between controller parameters and rotor dynamics. In conjunction with the GAUD, this approach improves the data efficiency and predication capacity relative to conventional surrogates and clarifies controller–plant interactions.
3. The integrated GRU-KAN-UD workflow replaces repeated finite element simulations with rapid surrogate evaluation, enabling fast parametric exploration and early-stage design studies, thereby lowering the computational burden without compromising modeling fidelity.

The remainder of this manuscript is organized as follows. Section 2 formulates the AMB–rotor dynamic model. Section 3 details the GAUD design and GRU-KAN surrogate methodology. Section 4 presents the numerical and experimental results. Finally, Section 5 summarizes our findings.

## 2. AMB System Model

**Figure 1:**
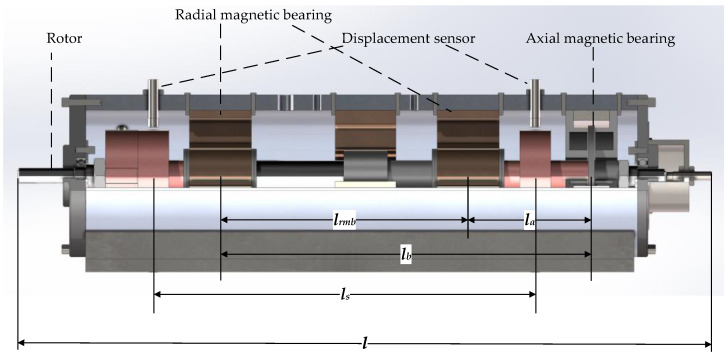
Structure diagram of AMB system.

The AMB–rotor system is a prototypical, inherently unstable system. Therefore, unlike conventional rotor dynamics, the modeling and dynamic analysis of AMB–rotors primarily focus on control issues. The controlled plant includes not only the rotor structure but also displacement-sensing elements and power amplification units. To analyze and study the dynamics of the AMB–rotor, a dynamic model of the AMB system is first established. Figure 1 shows the five-degree-of-freedom configuration of the AMB–rotor. The structural parameters of the AMB and rotor are listed in Table 1 and Table 2.

### 2.1. Construction of the AMB–Rotor Dynamic Model

The dynamic equation of the AMB system can be written as

(1) $$m\overset{\cdot \cdot }{x}+c_{e}\overset{\cdot }{x}+k_{e}x=f$$

where *m* is the rotor mass, *x* the displacement, *f* the force acting on the rotor, $c_{e}$ the equivalent damping coefficient, and $k_{e}$ the equivalent stiffness coefficient.

Taking the Laplace transform of Equation (1) under zero initial conditions yields the frequency-domain transfer function (with s=jω)

(2) $$\frac{X(j\omega )}{F(j\omega )}=\frac{1}{-m\omega^{2}+k_{e}+c_{e}j\omega }$$

where *s* denotes the Laplace variable, $j=\sqrt{-1}$ is the imaginary unit, and ω is the angular frequency.

In this paper, an eddy current sensor is selected as the displacement sensor due to its simple structure, compact size, and high sensitivity. Its transfer function is

(3) $$G_{s}\left(s\right)=A_{s}$$

where $A_{s}$ denotes the sensor gain.

Within the frequency response range, the power amplifier can be modeled as a first-order inertial element:

(4) $$G_{a}\left(s\right)=\frac{A_{a}}{T_{a}s+1}$$

where $A_{a}$ is the amplifier gain and $T_{a}$ its time constant.

The support characteristics of the AMBs are principally determined by the control system. Thus, an analysis of stiffness and damping must be carried out under a given control strategy. Different controller parameters or algorithms yield distinct dynamic support properties. The closed-loop transfer function in the frequency domain is given in [25]:

(5) $$\frac{X(j\omega )}{F(j\omega )}=\frac{1}{-m\omega^{2}+k_{xx}+k_{ix}G_{s}(j\omega )G_{a}(j\omega )G_{c}(j\omega )}$$

where $k_{xx}$ is the displacement–stiffness coefficient and $k_{ix}$ the current–stiffness coefficient, and $G_{c}\left(j\omega \right)$ denotes the controller transfer function.

The electromagnetic stiffness and damping coefficients are defined by

(6) $$\left\{\begin{array}{c} k_{xx}=-\frac{\mu_{0}n^{2}AI_{0}^{2}}{2x_{0}^{3}}\\ k_{ix}=\frac{\mu_{0}n^{2}AI_{0}}{2x_{0}^{2}} \end{array}\right.$$

with $\mu_{0}$ being the permeability of free space, *n* the coil turns, *A* the pole area, $I_{0}$ the bias current, and $x_{0}$ the one-side air gap.

By combining Equations (2) and (5), substituting Equations (3) and (4), and applying the incomplete differential PID algorithm, the expression for the dynamic support characteristics is obtained:

(7) ke=kxx+kixReGc(s)Gs(s)Ga(s)=kxx+kixReKp+Kis+Kds1+TdsAsAa1+Tas,ce=kixωImGc(s)Gs(s)Ga(s)=kixωImKp+Kis+Kds1+TdsAsAa1+Tas.

where $K_{p}$, $K_{i}$, $K_{d}$, and $T_{d}$ are the proportional, integral, derivative, and incomplete differential gains of the PID controller; Re{·} and Im{·} denote the real and imaginary parts of a transfer function, respectively.

**Figure 2:**
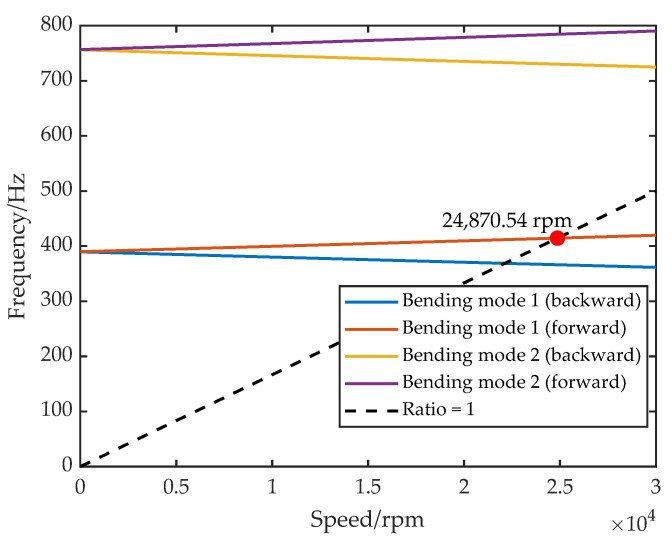
Campbell diagram of AMB–rotor.

Based on these equivalent stiffness and damping definitions, FEM was performed in Ansys Workbench. A nominal mesh size of 5 mm was used, yielding 92,442 nodes and 26,114 elements after meshing. Boundary conditions reflect the five-DOF configuration: the AMB supports were modeled as spring connections located at the bearing stations. Using the Routh stability criterion, parameters were chosen as $K_{p}=2000$, $K_{i}=0.1$, $K_{d}=20$, $T_{d}=1\times 10^{-4}$ and $I_{0}=1.4$ A, resulting in equivalent stiffness of $6.23\times 10^{6}$ N/m and damping of $4.33\times 10^{4}$ N·s/m. Gyroscopic effects were enabled for rotational speed sweeps to generate the Campbell diagram in Figure 2. As the speed increases, gyroscopic effects split the rotor’s characteristic frequencies: the forward whirl rises, while the backward whirl falls, yielding a first critical speed of 24,870.54 rpm.

### 2.2. Effects of Individual Control Parameters on Critical Speed

**Figure 3:**
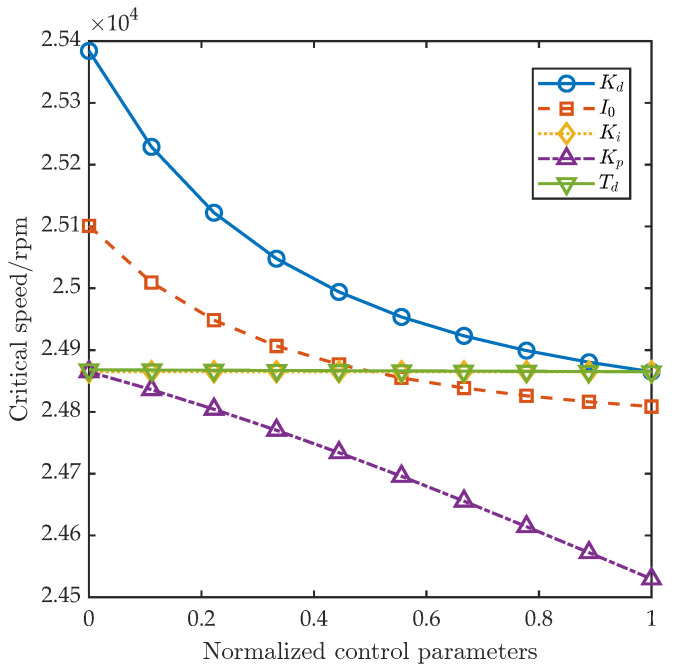
The influence of the control parameters on the dynamic characteristics of the AMB–rotor.

Based on the FEM, the influence of each single control parameter (proportional gain $K_{p}$, integral gain $K_{i}$, derivative gain $K_{d}$, incomplete differential gain $T_{d}$, and bias current $I_{0}$) on the AMB–rotor’s critical speed was investigated. Each parameter was divided into ten equally spaced levels over its range (Table 3), while the other parameters were held at their mean values. FEM yielded the normalized first-order critical speed curves versus each parameter, as shown in Figure 3. The analysis indicates that increasing $K_{p}$ significantly lowers the first critical speed; similarly, increases in $K_{d}$ and $I_{0}$ also reduce it, albeit with a more gradual trend than for $K_{p}$. By contrast, variations in $K_{i}$ and $T_{d}$ have negligible effects on the critical speed.

## 3. Surrogate Modeling of Control Parameters Versus Critical Speed

The direct sensitivity analysis of the control parameters is challenging due to the high dimensionality, strong parameter coupling, and substantial computation time. Surrogate models offer an efficient alternative, enabling the rapid evaluation of multivariable effects with significantly reduced complexity [26].

In this paper, we build a surrogate that maps the five control parameters ($K_{p}$, $K_{i}$, $K_{d}$, $I_{0}$, $T_{d}$) to the first critical speed. All FEM and surrogate model training was performed on a workstation with an Intel i9-9900 CPU (INTEL CHINA Co. Ltd., Shanghai, China) and 64 GB RAM (Shenzhen Kingbank Technology Co. Ltd., Shenzhen, China).

### 3.1. Surrogate Model Framework

The surrogate model construction follows two primary steps: the selection of initial samples and model training [27].

#### 3.1.1. Uniform Design Sampling

**Figure 4:**
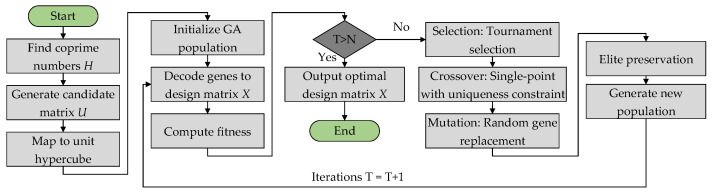
The flowchart of GAUD.

Traditional uniform design can produce anisotropic samples in high-dimensional spaces [28], while Latin hypercube sampling may miss the response surface curvature [24]. We introduce a genetic algorithm-enhanced uniform design (GAUD) to generate more representative samples [29]. The flowchart of GAUD can be seen in Figure 4.

For *n* experiments, compute the set of integers coprime to *n*,

(8) $$H=\left\{h_{1},\;h_{2},\;\ldots ,\;h_{m}\right\},\;m=\phi \left(n\right),$$

where ϕ(n) is Euler’s totient function. Form an n×m candidate matrix $U=[u_{ij}]$,

(9) $$u_{ij}\;=\;i\cdot h_{j}\;\mathrm{mod}\;n,\;i=1,\ldots ,n,\;j=1,\ldots ,m.$$

Map each entry to the midpoint of (0,1),

(10) $$x_{ij}\;=\;\frac{u_{ij}+0.5}{\;n\;},\;x_{ij}\in \left(0,1\right),$$

yielding *m* columns of factor levels. These samples initialize the genetic algorithm, guided by a composite bias measure that adapts through fitness-driven iterations to capture both sample diversity and parameter coupling.

The algorithm minimizes the mixed bias measure ${\mathrm{MD}}^{2}\left(x\right)$,

(11) $$\min_{x\in X_{n\times n_{f}}}{\mathrm{MD}}^{2}\left(x\right),$$

where $n_{f}$ is the number of factors and $X_{n\times n_{f}}$ the sample table. The measure is defined as

(12) $$\begin{array}{cc} MD^{2}\left(x\right) & ={\left(\frac{19}{12}\right)}^{n_{f}}-\\ \; & \frac{2}{n}\sum_{i=1}^{n}\prod_{j=1}^{n_{f}}\left(\frac{5}{3}-\frac{1}{4}\left|x_{ij}-\frac{1}{2}\right|-\frac{1}{4}{\left|x_{ij}-\frac{1}{2}\right|}^{2}\right)+\\ \; & \frac{1}{n^{2}}\sum_{i=1}^{n}\sum_{k=1}^{n}\prod_{j=1}^{n_{f}}\left(\begin{array}{c} \frac{15}{8}-\frac{1}{4}|x_{ij}-\frac{1}{2}|-\frac{1}{4}\left|x_{kj}-\frac{1}{2}\right|\\ -\frac{3}{4}|x_{ij}-x_{kj}|+\frac{1}{2}{\left|x_{ij}-x_{kj}\right|}^{2} \end{array}\right) \end{array}$$

#### 3.1.2. Support Vector Regression

SVR allows an error tolerance zone $\varepsilon_{s}$, penalizing only deviations beyond this threshold [30]. The prediction function is

(13) $$f\left(x\right)=v^{T}\phi \left(x\right)+b$$

where ϕ(x) is the nonlinear mapping function, *v* the weight vector, and *b* the bias term. SVR minimizes the regularized risk

(14) $$\min_{v,b,\{\xi_{i},{\hat{\xi }}_{i}\}}\;\frac{1}{2}{∥v∥}^{2}+C\sum_{i=1}^{m}\left(\xi_{i}+{\hat{\xi }}_{i}\right)$$

subject to

(15) $$\left\{\begin{array}{c} y_{i}-f\left(x_{i}\right)\leq \varepsilon_{s}+\xi_{i},\\ f\left(x_{i}\right)-y_{i}\leq \varepsilon_{s}+{\hat{\xi }}_{i},\\ \xi_{i},\;{\hat{\xi }}_{i}\geq 0, \end{array}\right.$$

where C>0 is a regularization parameter balancing model complexity and tolerance, and $\xi_{i},{\hat{\xi }}_{i}$ are slack variables. By applying Lagrange duality, the solution takes the form

(16) $$f\left(x\right)=\sum_{i=1}^{m}\left(\alpha_{i}-{\hat{\alpha }}_{i}\right)\;K\left(x,x_{i}\right)+b$$

where $\alpha_{i},{\hat{\alpha }}_{i}$ are Lagrange multipliers and K(·,·) is the kernel function mapping inputs to a high-dimensional feature space.

#### 3.1.3. Kriging

Kriging represents the response surface G(Θ) as the combination of a regression term and a Gaussian process [31]

(17) $$G\left(\Theta \right)=\sum_{i=1}^{p}\beta_{i}\;w_{i}\left(\Theta \right)+z\left(\Theta \right)$$

where $w_{i}\left(\Theta \right)$ denotes the chosen basis functions, $\beta_{i}$ denotes the regression coefficients fitted to the data, and $z\left(\Theta \right)∼N\left(0,\sigma^{2}\right)$ is a zero-mean Gaussian process capturing local deviations. The covariance between two sample points $\Theta^{\left(i\right)}$ and $\Theta^{\left(j\right)}$ is [32]

(18) $$Cov\left[z\left(\theta^{\left(i\right)}\right),z\left(\theta^{\left(j\right)}\right)\right]=\sigma^{2}\left[R\left(\theta^{\left(i\right)},\theta^{\left(j\right)}\right)\right]$$

where the matrix *R* encodes correlations critical to the prediction accuracy. The regression coefficients and process variance are estimated by

(19) $$\left\{\begin{array}{c} \hat{\beta }={\left(W^{T}R^{-1}W\right)}^{-1}W^{T}R^{-1}m_{k}\\ {\hat{\sigma }}^{2}={\left(m_{k}-W\hat{\beta }\right)}^{T}R^{-1}\left(m_{k}-W\hat{\beta }\right)/N_{T} \end{array}\right.$$

with $m_{k}$ being the vector of the observed responses and *W* the $N_{T}\times p$ regression matrix. The hyperparameters $\varepsilon ={\left[\varepsilon_{1},\ldots ,\varepsilon_{b}\right]}^{T}$ governing the covariance function are obtained by maximizing the log-likelihood:

(20) $$maxF\left(\varepsilon \right)=-\frac{N_{T}ln\left({\hat{\sigma }}^{2}\right)+ln\left|R\right|}{2}\left(\varepsilon ⩾0\right)$$

#### 3.1.4. Gated Recurrent Unit–Kolmogorov–Arnold Network

**Figure 5:**
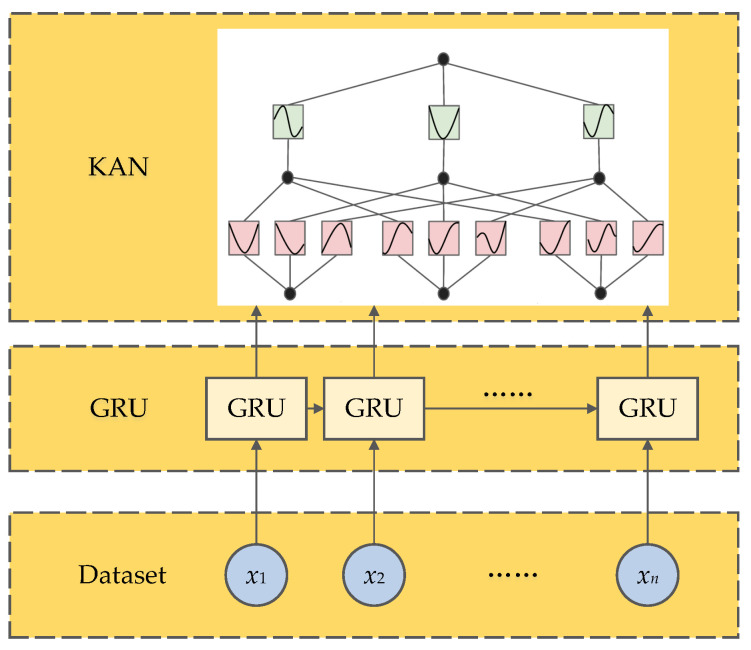
GRU-KAN network structure.

The GRU-KAN architecture combines gated recurrent units with Kolmogorov–Arnold networks to leverage the GRU’s temporal modeling and the KAN’s universal approximation, improving both the accuracy and interpretability [33]. Its structure is shown in Figure 5.

The GRU core updates are

(21) $$\left\{\begin{array}{c} r_{t}=\sigma \left(W_{r}x_{t}+U_{r}h_{t-1}+b_{r}\right)\\ z_{t}=\sigma \left(W_{z}x_{t}+U_{z}h_{t-1}+b_{z}\right)\\ {\tilde{h}}_{t}=\tanh\left(W_{h}x_{t}+U_{h}\left(r_{t}⊙h_{t-1}\right)+b_{h}\right)\\ h_{t}=\left(1-z_{t}\right)⊙h_{t-1}+z_{t}⊙{\tilde{h}}_{t} \end{array}\right.$$

where $z_{t}$ and $r_{t}$ are the reset and update gates; ${\tilde{h}}_{t}$ is the candidate state; $h_{t}$ is the hidden state; σ(·) is the sigmoid function; tanh(·) is the hyperbolic tangent; and $W_{*},U_{*},b_{*}$ are the weight and bias parameters.

The KAN decomposes a multivariate function into univariate components

(22) $$fx_{1},x_{2},\ldots ,x_{n}=\sum_{q=1}^{2n+1}\phi_{q}\left(\sum_{p=1}^{n}\psi_{p,q}(x_{p})\right)$$

where both $\phi_{q}\left(\cdot \right)$ and $\psi_{p,q}\left(\cdot \right)$ are one-dimensional continuous functions.

(23) $$x^{\left(l+1\right)}=\Phi^{\left(l\right)}\left(x^{\left(l\right)}\right),$$

with

(24) $$\Phi^{\left(l\right)}=\left(\begin{array}{cccc} \phi_{1,1}^{\left(l\right)}\left(\cdot \right) & \phi_{1,2}^{\left(l\right)}\left(\cdot \right) & \ldots & \phi_{1,n_{l}}^{\left(l\right)}\left(\cdot \right)\\ \phi_{2,1}^{\left(l\right)}\left(\cdot \right) & \phi_{2,2}^{\left(l\right)}\left(\cdot \right) & \ldots & \phi_{2,n_{l}}^{\left(l\right)}\left(\cdot \right)\\ \vdots & \vdots & \ddots & \vdots \\ \phi_{n_{l+1},1}^{\left(l\right)}\left(\cdot \right) & \phi_{n_{l+1},2}^{\left(l\right)}\left(\cdot \right) & \ldots & \phi_{n_{l+1},n_{l}}^{\left(l\right)}\left(\cdot \right) \end{array}\right)$$

where $n_{l}$ is the number of units in layer *l*, $x_{i}^{\left(l\right)}$ is the projection vector for unit *i*, and $\phi_{i,j}^{\left(l\right)}\left(\cdot \right)$ is the univariate activation function applied to its input.

### 3.2. Model Result Analysis

**Figure 6:**
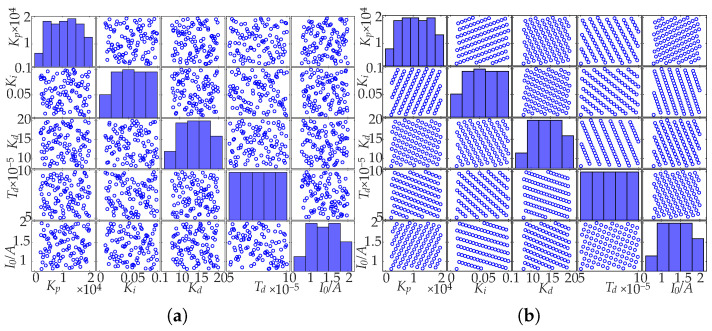
Sample scatter plots of (**a**) LHS and (**b**) GAUD.

**Figure 7:**
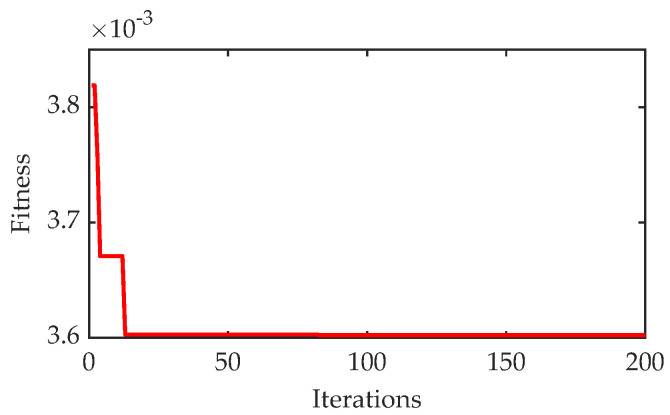
Convergence curve of GAUD.

At n=100 and $n_{f}=5$, the initial samples generated by LHS and GAUD are shown in Figure 6. For the GAUD sampling procedure, the population size is set to 30 and the number of iterations to 200. The convergence behavior under these settings is illustrated in Figure 7. Using the mixed bias measure from Equation (12), GAUD achieves ${\mathrm{MD}}^{2}\left(x\right)=0.0036$, significantly lower than LHS’s 0.012, indicating a more uniform sample space.

To demonstrate the feasibility and advantages of the GRU-KAN model built on GAUD (GRU-KAN-UD), we compare five mappings from the control parameters to the critical speed: GRU-KAN-LHS, Kriging-UD, Kriging-LHS, SVR-UD, and SVR-LHS. All surrogate inputs are min–max normalized to [0,1]. The hyperparameters for GRU-KAN, SVR, and Kriging are tuned via Bayesian optimization with five-fold cross-validation. The search spaces are summarized in Table 4.

**Figure 8:**
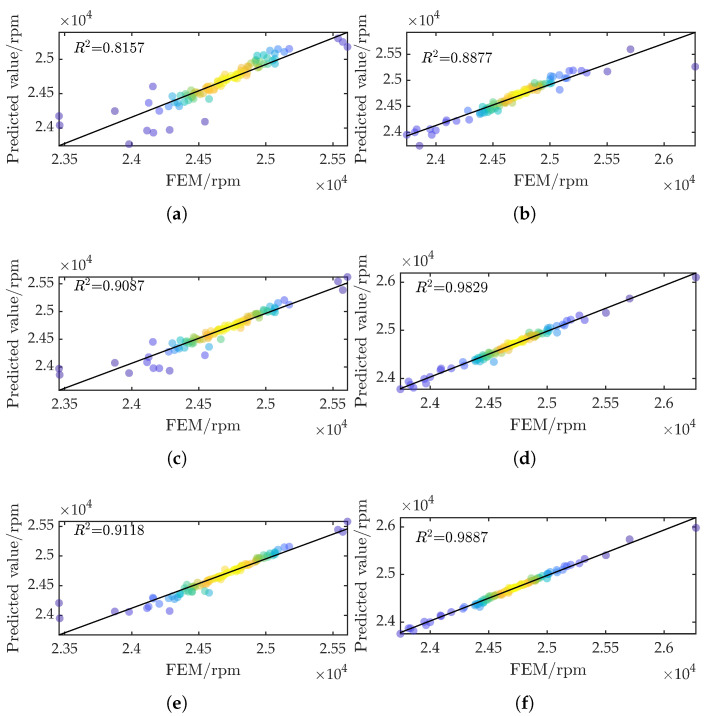
Comparison of residuals for first critical speed predictions: (**a**) SVR-LHS; (**b**) SVR-UD; (**c**) Kriging-LHS; (**d**) Kriging-UD; (**e**) GRU-KAN-LHS; (f) GRU-KAN-UD.

The residuals for the first critical speed are plotted in Figure 8. Compared with LHS sampling, Kriging-UD and GRU-KAN-UD maintain high confidence across both low and high critical speeds, confirming that GAUD yields a broader yet accurate sample space.

**Figure 9:**
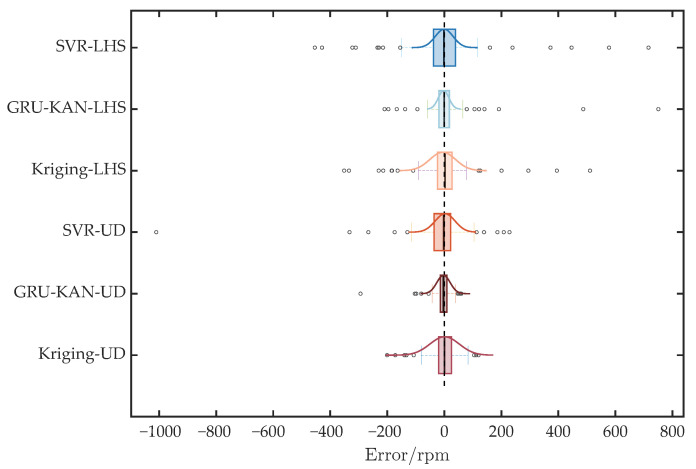
Comparison of error distributions under different models.

The error distributions are compared in Figure 9. All models exhibit approximately normal error distributions. GAUD sampling shows a smaller interquartile range than LHS sampling, indicating tighter error concentration and superior predictive performance; GRU-KAN-UD has the smallest range and interquartile spread.

For a quantitative evaluation, we compute the following metrics: mean absolute error ($E_{m}$), mean absolute percentage error ($E_{mp}$), mean square error ($E_{ms}$), and root mean square error ($E_{rms}$).

(25) $$E_{m}=\frac{1}{n}\sum_{i=1}^{n}\left|y_{i}-{\hat{y}}_{i}\right|,$$

(26) $$E_{mp}=\frac{100\%}{n}\sum_{i=1}^{n}\frac{\left|y_{i}-{\hat{y}}_{i}\right|}{y_{i}},$$

(27) $$E_{ms}=\frac{1}{n}\sum_{i=1}^{n}{\left(y_{i}-{\hat{y}}_{i}\right)}^{2},$$

(28) $$E_{rms}=\sqrt{\frac{1}{n}\sum_{i=1}^{n}{\left(y_{i}-{\hat{y}}_{i}\right)}^{2}}.$$

The coefficient of determination is

(29) $$R^{2}=1-\frac{\sum_{i=1}^{n}{\left(y_{i}-{\hat{y}}_{i}\right)}^{2}}{\sum_{i=1}^{n}{\left(y_{i}-\bar{y}\right)}^{2}},$$

where *n* is the number of samples, $y_{i}$ the observed value, ${\hat{y}}_{i}$ the predicted value, and $\bar{y}$ the sample mean.

The standard deviation is

(30) $$\mathrm{SD}=\sqrt{\frac{1}{N}\sum_{i=1}^{N}{\left({\hat{y}}_{i}-\bar{y}\right)}^{2}}.$$

The evaluation metrics are summarized in Table 5. GRU-KAN-UD delivers the lowest errors and the highest $R^{2}=0.9887$, indicating a highly reliable model (an $R^{2}$ above 0.9 denotes strong confidence). Furthermore, its SD most closely matches the actual SD ($3.78\times 10^{2}$ rpm).

To compare the relative performance across different metrics, we normalize each error measure $E_{i,j}$ (where j∈{m,mp,ms,rms} indexes $\;E_{m}$, $E_{mp}$, $E_{ms}$, $E_{rms}$ and i,k indexes the model) as

(31) $$E_{nom,i,j}=\frac{\frac{\max_{k}E_{k,j}}{E_{i,j}}}{\sum_{i=1}^{n}\frac{\max_{k}E_{k,j}}{E_{i,j}}}$$

and similarly normalize the coefficient of determination:

(32) $$R_{nom,i}^{2}=\frac{R_{i}^{2}}{\sum_{i=1}^{n}R_{i}^{2}}$$

**Figure 10:**
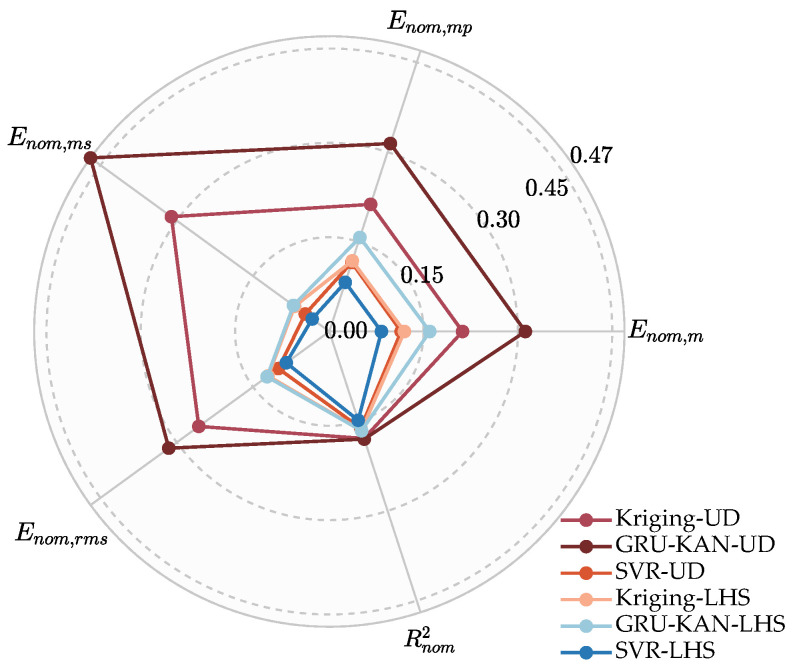
Radar chart of normalized metrics for different models.

The radar chart in Figure 10 visualizes each model’s composite performance, with a larger enclosed area indicating better overall metrics. GRU-KAN-UD notably outperforms all others, while SVR-UD’s normalized $E_{m}$ and $E_{mp}$ closely match those of Kriging-LHS, further confirming the advantage of GAUD sampling.

### 3.3. Analysis of Control Parameter Coupling and Critical Speed

Using the GRU-KAN-UD surrogate, we examine the response surfaces of the first critical speed as a function of the bias current $I_{0}$ and each control parameter.

**Figure 11:**
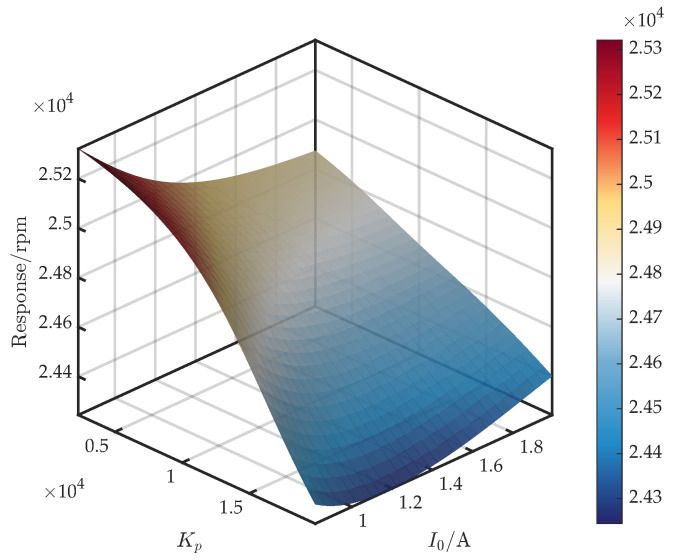
Response surface of first critical speed versus $I_{0}$ and $K_{p}$.

Figure 11 shows the surface for $\left(I_{0},K_{p}\right)$. As demonstrated in Equations (6) and (7), the parameters $k_{xx}$ and $k_{ix}$ are proportional to $I_{0}^{2}$ and $I_{0}$, respectively. The equivalent stiffness ke=kxx+kixReGc(·)Gs(·)Ga(·); thus, both $K_{p}$ (entering Re{Gc}) and $I_{0}$ strongly regulate $k_{e}$ and, in turn, the critical speed. Furthermore, across the full $I_{0}$ range, the critical speed decreases monotonically with $K_{p}$, with the rate of decline inversely related to $I_{0}$. For $K_{p}\in \left[2000,11,000\right]$, the critical speed also decreases monotonically with $I_{0}$; for $K_{p}\in \left(11,000,20,000\right]$, the response to $I_{0}$ is non-monotonic—initially decreasing and then rising. A comparison with Figure 3 reveals that the FEM trends hold only at a specific $K_{p}$, demonstrating the surrogate’s value in capturing full coupling effects.

**Figure 12:**
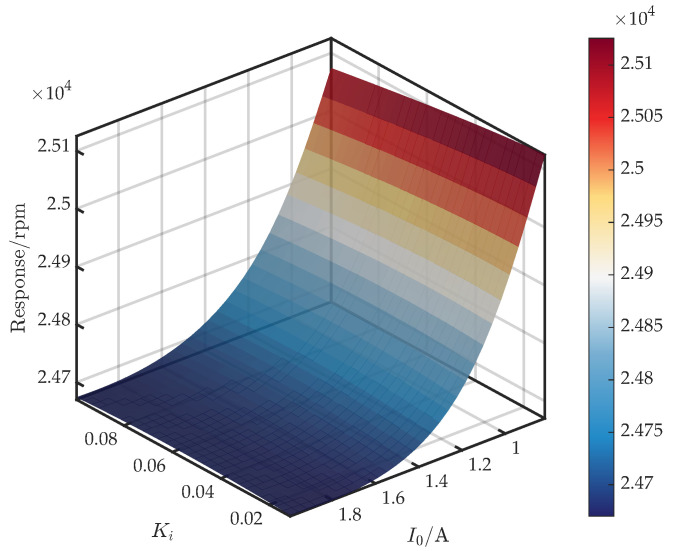
Response surface of first critical speed versus $I_{0}$ and $K_{i}$.

Figure 12 shows the surface for $\left(I_{0},K_{i}\right)$. From Equation (7), $K_{i}$ contributes mainly at low frequencies to Im{Gc}, so its effect on $k_{e}$ and $c_{e}$ is limited within the operational bandwidth. Consequently, the influence of $K_{i}$ on the critical speed is expected to be weak. Furthermore, when $I_{0}\in \left[0.8,1.3\right]$, the critical speed decreases monotonically with $K_{i}$. For $I_{0}\in \left(1.3,2\right]$, the response is non-monotonic: it increases slightly before decreasing.

**Figure 13:**
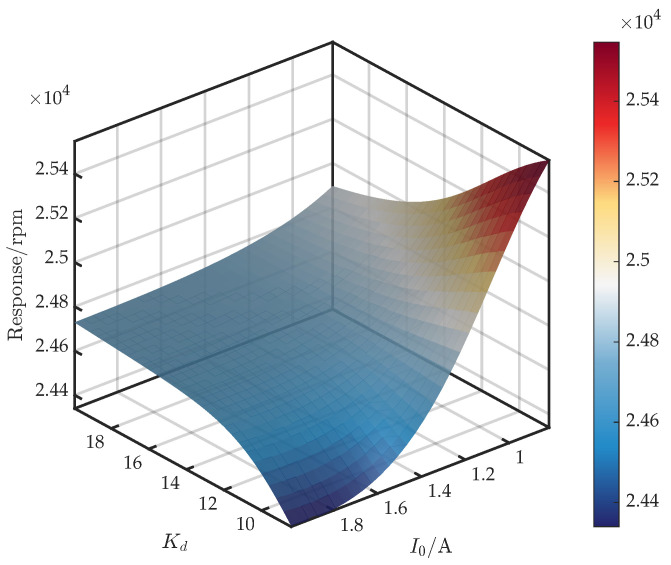
Response surface of first critical speed versus $I_{0}$ and $K_{d}$.

Figure 13 shows the surface for $\left(I_{0},K_{d}\right)$. From Equations (6) and (7), $K_{d}$ amplifies Im{Gc} in the relevant band, so $K_{d}$ directly increases $c_{e}$, while $I_{0}$ scales with $k_{ix}$ and simultaneously affects $c_{e}$. Moreover, because $I_{0}$ exerts an influence on both $k_{e}$ and $c_{e}$, its net impact on the critical speed is stronger than that of $K_{d}$. Accordingly, for $I_{0}\in \left[1.1,2\right]$, the critical speed increases with $K_{d}$. For $I_{0}\in \left[0.8,1.1\right)$, the response is non-monotonic: it rises and then falls.

**Figure 14:**
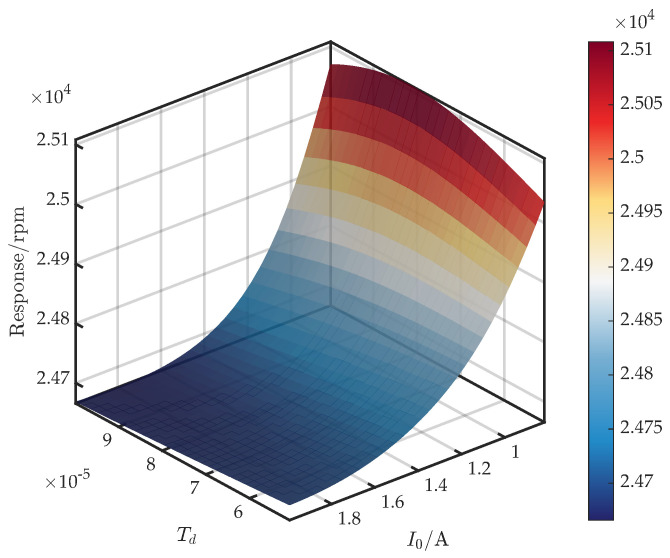
Response surface of first critical speed versus $I_{0}$ and $T_{d}$.

Figure 14 shows the surface for $\left(I_{0},T_{d}\right)$. From Equation (7), $T_{d}$ exerts a significant influence on the derivative filter, while contributing marginally to $k_{e}$ and $c_{e}$ within the operational frequency range; hence, a limited effect on the critical speed is expected. Accordingly, when $I_{0}\in \left[0.8,1.1\right]$, the critical speed exhibits a non-monotonic increase and then decrease with $T_{d}$. For $I_{0}\in \left(1.1,2\right]$, it decreases monotonically as $T_{d}$ increases.

## 4. Experimental Validation

**Figure 15:**
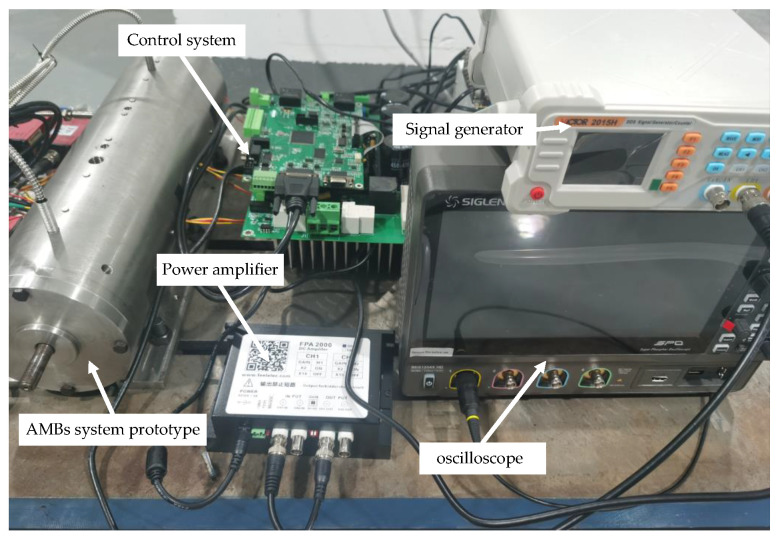
Schematic diagram of the test platform.

An acceleration test was performed to validate the AMB–rotor surrogate model based on GRU-KAN-UD. A PID controller with gains $K_{p}=2000$, $K_{i}=0.1$, $K_{d}=20$, $T_{d}=1\times 10^{-4}\;$ and bias current $I_{0}=1.4\;$ A was implemented to maintain stable levitation. The test platform schematic is shown in Figure 15.

**Figure 16:**
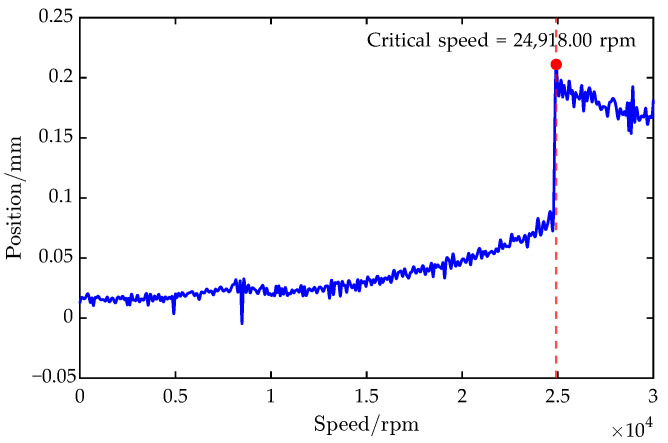
Experimental results of the AMB–rotor vibration responses.

Starting from rest, the rotor was accelerated at 60 rpm/s up to 30,000 rpm. The first critical speed was determined to be 24,918.00 rpm from the vibration response during acceleration, as can be seen in Figure 16.

Table 6 compares the first critical speeds obtained from the surrogate model, the FEM, and the experimental measurement. The FEM’s prediction deviated from the experiment by 47.46 rpm ($1.90\times 10^{-1}\;\%$), confirming its accuracy and providing a solid foundation for surrogate construction. The surrogate model’s prediction differed from the experiment by only 51.92 rpm ($2.08\times 10^{-1}\;\%$), demonstrating close agreement among the model, simulation, and test.

In terms of computation time, the surrogate evaluation required only $1.14\times 10^{-4}\;$ s versus 201 s for FEM—an improvement of over four orders of magnitude. This indicates that, once trained, the surrogate could rapidly yield key dynamic parameters without further simulation or experiments.

**Figure 17:**
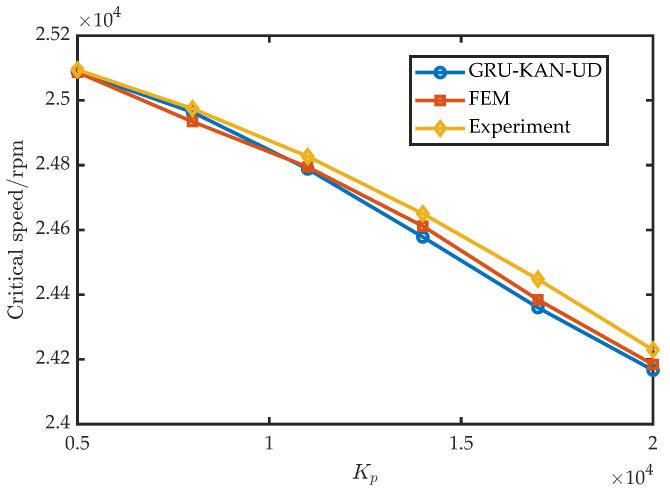
Experimental validation under varying $K_{p}$.

**Figure 18:**
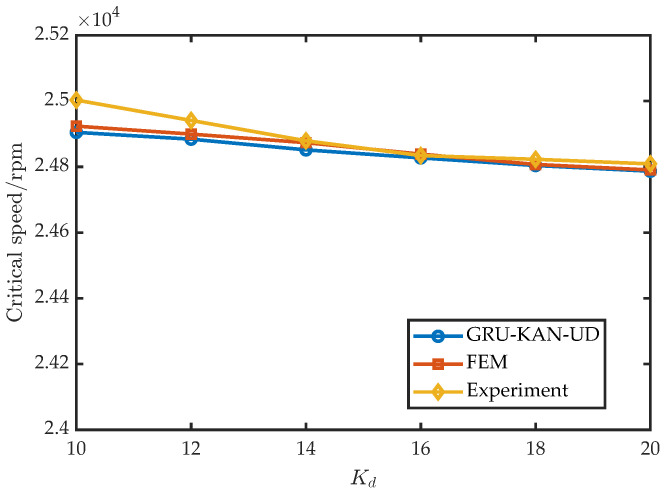
Experimental validation under varying $K_{d}$.

**Figure 19:**
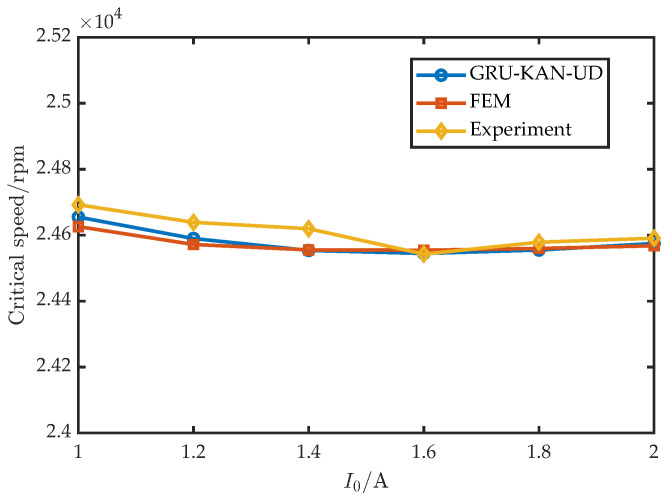
Experimental validation under varying $I_{0}$.

To assess the robustness across control settings, six values of $K_{p}$ were tested with $I_{0}=1\;$ A, $K_{i}=0.1$, $K_{d}=14$, $T_{d}=1\times 10^{-4}\;$; the results are shown in Figure 17. Similarly, six values of $K_{d}$ were tested with $I_{0}=1\;$ A, $K_{i}=0.1$, $K_{p}=$ 10,000, $T_{d}=1\times 10^{-4}\;$; the results appear in Figure 18. Furthermore, six values of $I_{0}$ were tested with $K_{d}=16\;$ A, $K_{i}=0.1$, $K_{p}=$ 15,000, $T_{d}=1\times 10^{-4}\;$; the results are shown in Figure 19.

Across 18 parameter combinations, the surrogate maintained high accuracy over the design space: relative to the experiment, $E_{m}=38.51$ rpm and $E_{mp}=1.56\times 10^{-1}\;\%$; relative to FEM, $E_{m}=14.30$ rpm and $E_{mp}=5.80\times 10^{-2}\;\%$.

## 5. Conclusions

The key findings are as follows:

1. We introduce a surrogate-based method for the analysis of the coupling between the control parameters and the AMB–rotor’s first critical speed. The GRU-KAN-UD surrogate achieves a mean absolute error of 38.51 rpm and a mean absolute percentage error of $1.56\times 10^{-1}$%. The experimental results confirm the surrogate’s feasibility and precision, providing a solid theoretical and practical foundation for AMB–rotor dynamic modeling.
2. Compared with FEM, the trained surrogate model reduces the computation time from 201 s to $1.14\times 10^{-4}\;$ s while maintaining high fidelity, demonstrating a significant speed advantage.
3. Unlike single-parameter studies, the surrogate approach captures the interaction effects among controller gains, offering a more comprehensive dynamic analysis.
4. We propose a genetic algorithm-enhanced uniform design for sample selection, improving the robustness of surrogate training. The resulting GRU-KAN-UD attains the highest goodness of fit among all candidates ($R^{2}=0.9887$) and the lowest mean absolute error in cross-validation (22.06 rpm), with error reductions of over 60% relative to Kriging-LHS and over 70% relative to SVR-LHS.

The GRU-KAN surrogate is tailored to AMB–rotor dynamics and performs well on the studied configuration. However, its ability to generalize across other fields may be limited. Future work may explore ensemble learning and domain adaptation to enhance the robustness and out-of-domain generalization.

The present study focuses on predicting the critical speed; transient amplitude prediction under time-varying conditions remains to be explored. To broaden the applicability in magnetic bearing rotors, future extensions should incorporate external loads and system condition variations such as temperature rises.

The surrogate provides practical utility for controller pre-tuning and for design space exploration from a rotor dynamics perspective, enabling efficient early-stage assessment without repeated high-fidelity simulations.

## Figures and Tables

**Table 1:** Basic parameters of the radial AMB.

Parameter	Value
Bias current (A)	1.4
Number of coil turns	171
Pole area (mm^2^)	742
One side air gap (mm)	0.8

**Table 2:** Basic parameters of the AMB–rotor.

Parameter	Variable	Value
Rotor mass (kg)	*m*	4.3
Total rotor length (mm)	*l*	502
Distance between radial AMB centers (mm)	$l_{1}$	192
Distance between end sensor centers (mm)	$l_{s}$	296
Right AMB to thrust disk distance (mm)	$l_{a}$	287.5
Left AMB to thrust disk distance (mm)	$l_{b}$	95.5

**Table 3:** Ranges of control parameters.

Control Parameter	Range
$K_{p}$	[2000, 20,000]
$K_{i}$	[0.01, 0.1]
$K_{d}$	[8, 20]
$T_{d}$	[$5\times 10^{-5}$, $1\times 10^{-4}$]
$I_{0}$ (A)	[0.8, 2]

**Table 4:** Hyperparameter search spaces.

Model	Hyperparameter	Range/Options
GRU-KAN	Batch size	{16, 32, 64, 128}
	Learning rate	$\left[10^{-4},10^{-1}\right]$
	Hidden dimension	integers [16,64]
	GRU layers	integers [1,3]
SVR	Kernel type	{linear, poly, rbf, sigmoid}
	Regularization strength	[0.1,100]
	Epsilon tube width	[0.01,1.0]
Kriging	Kernel type	{rbf, matern}
	Length scale	$\left[10^{-3},10^{3}\right]$
	Constant value	$\left[10^{-3},10^{3}\right]$
	Noise level	$\left[10^{-10},10^{-3}\right]$

**Table 5:** First critical speed prediction error.

Method	$E_{m}$ (rpm)	$E_{\mathrm{mp}}$ (%)	$E_{\mathrm{ms}}$	$E_{\mathrm{rms}}$ (rpm)	$R^{2}$	SD (rpm)
SVR-LHS	83.03	$3.40\times 10^{-1}$	$2.26\times 10^{4}$	150.31	0.8157	$2.96\times 10^{2}$
SVR-UD	60.21	$2.42\times 10^{-1}$	$1.61\times 10^{4}$	126.97	0.8877	$3.14\times 10^{2}$
Kriging-LHS	57.60	$2.36\times 10^{-1}$	$1.12\times 10^{4}$	105.77	0.9087	$3.31\times 10^{2}$
Kriging-UD	32.47	$1.31\times 10^{-1}$	$2.46\times 10^{3}$	49.58	0.9829	$3.63\times 10^{2}$
GRU-KAN-LHS	43.06	$1.78\times 10^{-1}$	$1.08\times 10^{4}$	103.97	0.9118	$3.03\times 10^{2}$
GRU-KAN-UD	22.06	$8.88\times 10^{-2}$	$1.63\times 10^{3}$	40.32	0.9887	$3.63\times 10^{2}$

**Table 6:** Comparison of the first critical speeds of various models.

Model/Test Type	Critical Speed (rpm)	$E_{m}$ (rpm)	$E_{\mathrm{mp}}$(%)
GRU-KAN-UD	24,866.08	51.92	$2.08\times 10^{-1}$
FEA	24,870.54	47.46	$1.90\times 10^{-1}$
Acceleration test	24,918.00	-	-

## Data Availability

Data will be made available by the corresponding author on request.
